# NMR and Rheological Study of Anion Size Influence on the Properties of Two Imidazolium-based Ionic Liquids

**DOI:** 10.1038/s41598-017-09509-2

**Published:** 2017-08-21

**Authors:** Stephen M. Green, Michael E. Ries, Jamie Moffat, Tatiana Budtova

**Affiliations:** 1 Soft Matter Physics Research Group, School of Physics and Astronomy, University of Leeds, Leeds, LS2 9JT United Kingdom; 2 Innovia Films R&D Centre, West Road, Wigton, Cumbria CA7 9XX United Kingdom; 3 MINES ParisTech, PSL Research University, Centre for Material Forming (CEMEF), UMR CNRS 7635, CS 10207, 06904 Sophia Antipolis Cedex, France

## Abstract

NMR self-diffusion and relaxation, coupled with viscosity, were used to study the properties and structure of two imidazolium-based ionic liquids, 1-ethyl-3-methylimidazolium acetate [C_2_MIM][OAc] and 1-ethyl-3-methylimidazolium octanoate [C_2_MIM][OOct]. The experimental results point to the formation of different types of aggregates in each ionic liquid. These aggregates are small and stable under flow and temperature in [C_2_MIM][OAc], whereas the aggregates are large and sensitive to flow and temperature in [C_2_MIM][OOct]. In the latter case the size of aggregates decreases both under flow and temperature increase.

## Introduction

Ionic liquid (IL) is the term used to describe a salt that is in a liquid state below 100 °C. ILs have found many applications in a variety of fields, including biomass extraction and dissolution^1^, electrolytes^2, 3^, biodiesel production^4^, CO_2_ capture^5^, as well as in more novel uses such as Liquid Crystal Devices^6^ and medical research into viruses^7^. The field continues to grow rapidly, with thousands of new papers each year, reporting a wide range of ILs, their synthesis, properties and applications^8^.

One of the recent research directions in the field of ILs is their use as cellulose solvents^9–17^. Cellulose, the main component of plant cell walls, is the most abundant biopolymer on the planet. As such, it has a huge potential as a renewable material and chemical feedstock. The development of an efficient, environmentally friendly and commercially viable process for the utilisation of cellulose from plant biomass and further processing has become somewhat of a ‘Holy Grail’ in the field of Green Chemistry^18^. The relative insolubility of cellulose in common industrial solvents is the biggest obstacle to this and is mainly due to the formation of large hydrogen-bonded networks between cellulose chains^19^. Though, it is also thought that the amphiphilicity of cellulose may be a significant factor in its insolubility in most traditional solvents^20, 21^.

The early IL-cellulose studies considered mainly 1-alkyl-3-methylimidazolium (C_n_MIM) cations (*n* = 4–8) and a variety of anions, finding [C_4_MIM]Cl (*n* = 4) to be most effective at dissolving cellulose in their first set of trialed ILs^10, 22, 23^. It was shown that the length of the cation side-chain had significant effects on the properties of the IL and its ability to dissolve cellulose. Schubert *et al*. studied ILs with various [C_n_MIM]^+^ cations and found an odd-even cation chain length effect on solubility, with *n* = 4 showing the optimum solubility for a range of anions^24, 25^. Ismail *et al*. continued this line of study, using molecular dynamics calculations to investigate the dissolution of cellulose in C_n_MIM-based ILs^26, 27^. They analysed the bonding patterns between the cations, anions and cellulose chains. It was found that increasing the cation chain length caused the dynamics of the system to slow slightly but had only a minor effect on the solvation process.

In 2006, Fukaya *et al*.^28^ showed that formate anions, combined with imidazolium cations, result in low viscosity ILs and efficient dissolution of cellulose. One of the interesting features in imidazolium-based cations and carboxylate anions (for example, formate and acetate), is that the ion length can be varied in both the anion and the cation. [C_2_MIM][OAc] being liquid at room temperature with moderate viscosity, attracted a lot of interest with a huge number of publications regarding the properties and processing of these cellulose solutions^11, 13, 14, 16, 17, 29^. Xu *et al*. used the [C_4_MIM]^+^ cation with various anion functional groups, including formate and acetate, and found that cellulose solubility was improved by having electron donating groups (such as CH_2_ and CH_3_) attached to the carboxylate group^30^. In a subsequent paper, the C_4_MIM carboxylates were studied further to compare various thermodynamic properties with solubility data^31, 32^. They used [COOC_*m*−1_H_2(*m*−1)+1_] anions, finding that C_4_MIM propionate (*m* = 3) was the most effective at dissolving cellulose. This was hypothesised to be due to a favourable balance between increased solubility from the higher electron donating power of longer chains and decreased solubility due to steric hindrance, also because of the longer chains. Though, it should be noted that computational work by King *et al*. showed that the proton affinities, and therefore electron donating power, were very similar for the propionate and acetate anions^33^. Zhao *et al*. performed a systematic study of cellulose solubility with 3 imidazolium cations and 9 carboxylate anions^34^. It was found that C_2_MIM propionate was the most effective at dissolving cellulose, closely followed by C_2_MIM iso-butyrate. This supports the findings of Xu *et al*. that the electron donating power of the side chain is a key property in cellulose dissolution. There are many other factors affecting the suitability and efficiency of different ILs for cellulose dissolution.

One of the disadvantages of ILs is their high viscosity, which is undesirable for polymer dissolution and processing. One simple way to overcome this problem is to increase the dissolution temperature. Many IL-cellulose studies report that the dissolution process shows significant temperature dependence^10, 30, 34, 35^. This could be due to two main reasons, a thermodynamic barrier or dissolution kinetics. Recently, Andanson *et al*. measured the heat of dissolution for low concentrations of cellulose in [C_2_MIM][OAc], finding it to be strongly exothermic^36^. This suggests kinetic factors are actually dominating. The same was concluded from rheological studies of cellulose-[C_2_MIM][OAc] solutions: the thermodynamic quality of [C_2_MIM][OAc] decreases as temperature increases, meaning that elevated temperature is unfavorable in terms of cellulose-solvent interactions^29^. However, it is worth noting that recently, Minnick *et al*. showed an increase in cellulose solubility with increasing temperature in 1-ethyl-3-methylimidazolium diethyl phosphate^37^, suggesting some more complex factors at work. Though, the thermal behavior of the IL itself, together with potential cellulose degradation during dissolution for long periods at high temperatures (above 100 °C), are important factors to consider in cellulose dissolution studies^38, 39^.

Given the importance of solution viscosity in the dissolution process, a useful strategy for finding improved solvents would be to minimise the viscosity whenever possible. Due to the complex nature of IL aggregation, the obvious solution of choosing the smallest ions may not always be the most effective approach. Parviainen *et al*. studied a range of cations with both acetate and propionate anions^40^. In all possible cases, it was found that ILs with the propionate anion had a lower viscosity than the corresponding IL with an acetate anion. While only limited to anion chain lengths of 2 and 3, this trend hints at a contrast to the influence of the cation chain lengths on IL viscosity; the increase of cation length has been shown to increase viscosity steadily as *n* increases^41, 42^.

The Watanabe group did a series of systematic studies into the physicochemical properties of ILs^43–45^. Self-diffusion coefficients, viscosity and conductivity were measured for 3 series’ of ILs: first changing the anion^43^, then changing the cation chain length^44^ and then changing the cation entirely^45^. They found discrepancies when relating the properties to each other using the Stokes-Einstein and the Nernst-Einstein equations. In particular, when relating the experimentally measured conductivity to the conductivity calculated from experimental diffusion values, the diffusion-calculated conductivity was found to be too high in all ILs. The degree of the discrepancy depended on the identity of both the cation and anion and was attributed to ion clustering and aggregation^43^. Interestingly, in the case of changing the cation chain length, the longer chains led to more discrepancy and therefore a higher degree of aggregation was suggested^44^. Mass spectrometry was also used, showing the formation of multi-ion clusters^43^.

Wang and Voth used a coarse-graining computational method to investigate [C_n_MIM][NO_3_] ILs and how the cation tail length affects the microscopic structuring^46, 47^. They found that for ILs with n ≥ 3, the hydrophobic tail groups aggregated to form distinct domains, subject to the positioning of the charged head groups from Coulomb interactions with anions. These hydrophobic domains were stable, even up to 1200 K, and diffuse through the medium without losing their ordering.

Anion chain lengths of *m* = 1–4 have been studied previously^30–32, 34^; however, longer anion chain lengths have remained relatively unexplored. Longer cation chains have been shown to induce aggregation^48^, therefore, it would be expected that similar effects will be observed when changing the anion chain length.

**Figure 1:**
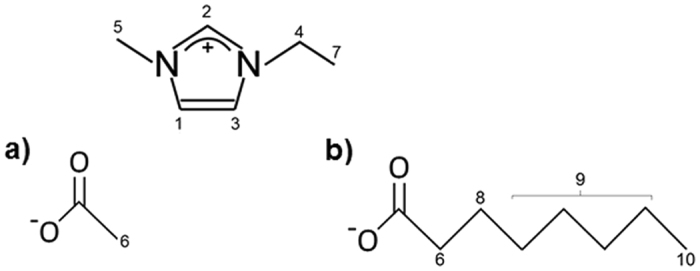
The two ionic liquids investigated in this study: (**a**) [C_2_MIM][OAc] and (**b**) [C_2_MIM][OOct]. NMR ^1^H resonances are labelled according to spectral assignment and previous publications^16^.

This paper focusses on the fundamental understanding of the influence of anion size on the properties of two imidazolium-based ionic liquids, 1-ethyl-3-methylimidazolium acetate [C_2_MIM][OAc] and 1-ethyl-3-methylimidazolium octanoate [C_2_MIM][OOct] (Fig. 1). A chain length of *m* = 8 in [C_2_MIM][OOct] was considered long enough to be significantly different to chains of *m* = 2 in [C_2_MIM][OAc] but not too long to cause viscosity problems when potentially dissolving cellulose. An extended NMR study (pulsed-field gradient diffusion measurements, relaxometry) was coupled with viscometry. Self-diffusion coefficients of all species were analysed using Stokes-Einstein-Debye approach.

## Results

### Self-diffusion

The ^1^H spectra for both ionic liquids are shown in Figure S1 in the Supplementary Information. Our previous work has shown that the chemical shift of peak 5 remains stable over the temperature range studied^14, 49^, therefore this peak was used as a calibration point for chemical shift changes *Δδ* as a function of temperature. The temperature dependence of *Δδ* is shown in Figure S2 in the Supplementary Information and is consistent with the expected bonding between the carboxylate anion and the hydrogen atoms on the imidazolium ring: peaks 1-3 have an upfield shift with increasing temperature. [C_2_MIM][OAc] shows equal temperature dependence in all three ring hydrogens (1, 2 and 3, see Figure S2a). In contrast, [C_2_MIM][OOct] shows a weaker temperature dependence for peak 2 (Figure S2b), indicating a stronger intermolecular hydrogen-bond for this hydrogen.

**Figure 2:**
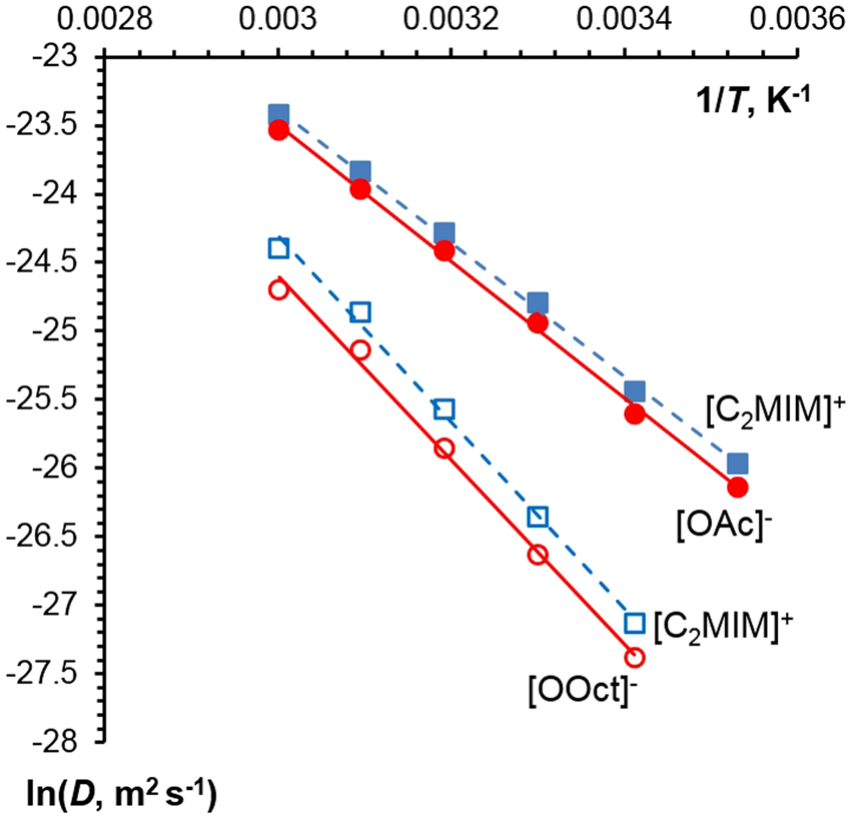
Self-diffusion coefficients for the cation and anion of both ILs as a function of the inverse temperature. Lines show linear fits. Uncertainty in *D* of 5% is approximately the size of points and has been omitted for clarity.

Figure 2 shows the variation of each ion’s diffusion coefficient with temperature. As expected, both ions in both ILs show increasing diffusion coefficients with increasing temperature, with the ions in the less-viscous [C_2_MIM][OAc] possessing higher *D* values than those in [C_2_MIM][OOct] (see viscosities in Figure S3 in the Supplementary Information). [C_2_MIM][OAc] shows the previously reported “anomalous” diffusion^13, 50^, i.e. the geometrically larger imidazolium cation diffusing faster than the smaller acetate anion (Fig. 2). One of the potential reasons for this slower-than-expected anion diffusion is ionic aggregation, in particular, anion-rich aggregates, as suggested in ref. 51.

According to the Stokes-Einstein equation, the diffusion coefficient is inversely proportional to the hydrodynamic radius. An estimation of the hydrodynamic radius of a molecule can be made from the following equation^52^,

1 $${r}_{h}=\frac{1}{2}{(\frac{M}{\rho {N}_{A}})}^{\frac{1}{3}}$$

where *M* is the molar mass, *ρ* is the density and *N*_*A*_ is the Avogadro number. For example, at 40 °C Eq. 1 gives *r*_*h*_ = 2.77 Å and 2.24 Å for the cation and anion of [C_2_MIM][OAc], respectively. The radii of the cation and anion at 40 °C in [C_2_MIM][OOct] are 2.86 Å and 3.11 Å, respectively.

Figure 2 shows that in [C_2_MIM][OOct] the larger anion diffuses experimentally slower than the cation, therefore diffusion is not anomalous, unlike in [C_2_MIM][OAc]. However, it should be noted that the theoretical ratio of the ionic radii of [C_2_MIM]^+^ to [OOct]^−^ is 0.92 (based on Eq. 1), whereas the experimental ratio of diffusion coefficients [C_2_MIM]^+^ to [OOct]^−^ is lower, around 0.75. Even though the [OOct]^−^ ion diffuses slower than the cation, as expected from the theoretical radii, it is experimentally diffusing yet slower than expected, which also points to the formation of anion-rich aggregates.

### Activation energy

Activation energies provide important insight into transport processes. Activation energies for molecular self-diffusion *E*_*a,D*_ were calculated using the results presented in Fig. 2 by fitting them to an Arrhenius-type equation,

2 $$lnD=ln{D}_{0}-\frac{{E}_{a,D}}{RT}$$

where *D*_0_ is a constant, *T* is temperature and *R* is the universal gas constant. An Arrhenius approach was also applied to viscosity data,

3 $$\mathrm{ln}\,\eta =ln{\eta }_{0}+\frac{{E}_{a,\eta }}{RT}$$

where *η* is Newtonian viscosity, *η*_0_ is a constant and *E*_*a,η*_ is the activation energy of viscous flow.

For the viscosity data, all flow curves showed a Newtonian plateau at least for two decades of shear rates, from 1 to 100 s^−1^ (See Figure S3a,b for [C_2_MIM][OAc] and [C_2_MIM][OOct], respectively, in the Supplementary Information). The mean values were taken for further analysis with standard deviations of less than 10%.

**Figure 3:**
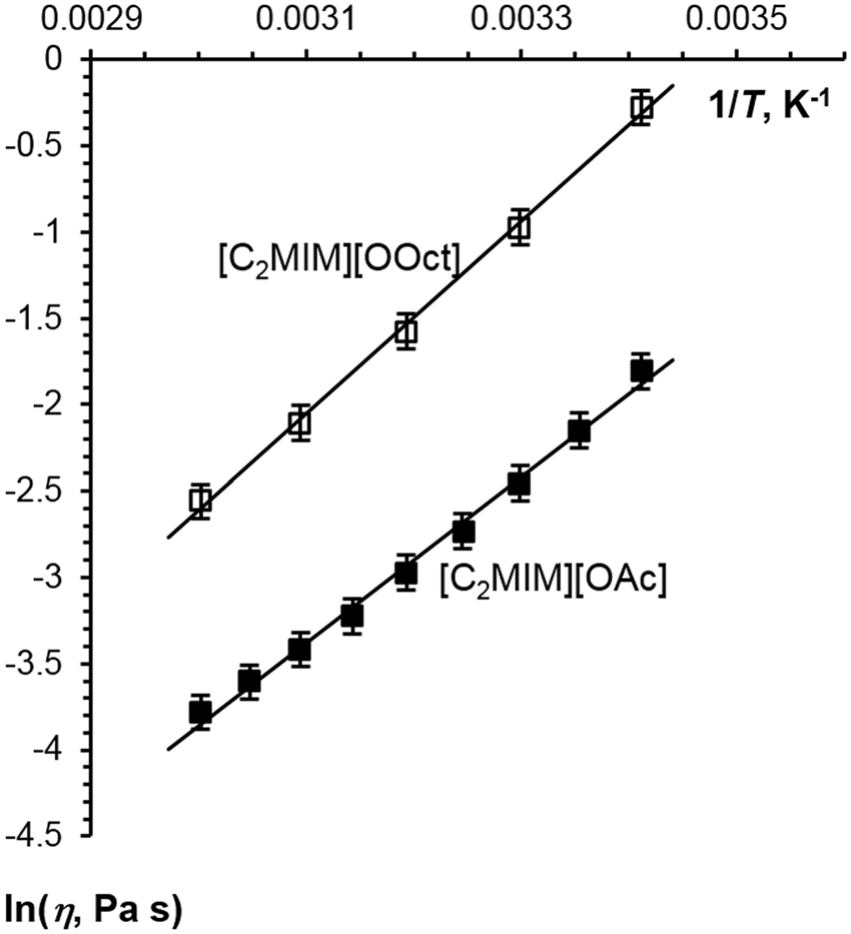
Newtonian viscosity as a function of inverse temperature for [C_2_MIM][OOct] and [C_2_MIM][OAc]. Lines are linear fits.

Viscosity-inverse temperature dependences are shown in Fig. 3; for both ILs the plots are linear in the range of temperatures studied. Each IL displays a decreasing viscosity as temperature increases, with [C_2_MIM][OOct] having a higher viscosity than [C_2_MIM][OAc] at all temperatures studied. This is as expected, with the higher molecular weight and longer chain of the octanoate anion increasing the overall viscosity. The corresponding *E*_*a,η*_ and *E*_*a,D*_ values are given in Table 1.

**Table 1:** Activation energies for self-diffusion, for both ions in both ILs, and for viscous flow for both ILs.

Activation Energy, kJ mol^−1^	[C_2_MIM][OAc]	[C_2_MIM][OOct]
*E*_*a, D*_ (cation)	40.6 ± 0.8	57 ± 2
*E*_*a, D*_ (anion)	41.5 ± 0.8	56 ± 2
*E*_*a, η*_	40 ± 1	46 ± 1

Table 1 shows that the activation energy values are higher for [C_2_MIM][OOct] than those of [C_2_MIM][OAc], regardless of the experimental technique used. The higher molecular weight of [C_2_MIM][OOct] makes the translational motion required for both diffusion and viscosity more difficult to activate, giving a larger energy barrier for motion. The difference between cation diffusion activation energies for [C_2_MIM][OAc] and [C_2_MIM][OOct] reveals that a longer tail on the anion not only slows the anion down but also makes the energy barrier to movement larger for the cation. The activation energy values of [C_2_MIM][OAc] obtained from viscosity and diffusion measurements are the same, within experimental uncertainties, so they follow a Stokes-Einstein relationship^13^, as will be shown later. Interestingly, for [C_2_MIM][OOct] the value for diffusional activation energy is significantly higher than that of viscosity. *E*_*a,D*_ is indicative of the energy barrier for one molecule to diffuse from one position to another and depends on local microscopic interactions^53^. *E*_*a,η*_ represents the mean energy required for viscous flow in the medium and is related to the macroscopic frictional force of the system. We suggest that the difference between *E*_*a,D*_ and *E*_*a,η*_ in [C_2_MIM][OOct] is because shear flow may destroy, at least partially, the aggregates formed between ions, while the NMR recorded diffusion is “static” (without any shear stresses) and thus preserves the aggregates. This result is thus the first indication that aggregates formed in [C_2_MIM][OOct] are unstable, being disrupted by shear.

### Stokes-Einstein analysis and radii of ions

The well-known Stokes-Einstein equation correlates the self-diffusion coefficient of a component *i* with its effective hydrodynamic radius *r*_*h,i*_, zero shear rate viscosity and temperature,

4 $$D=\frac{{k}_{B}T}{6f\pi \eta {r}_{h,i}}$$

where *k*_*B*_ is the Boltzmann constant and *f* is a correction term^54–56^. In classical Brownian motion, where the diffusing sphere is large relative to the particles of the diffusing medium, *f* is equal to 1. However, if the diffusing particle is small or similarly sized to the surrounding diffusing molecules, then *f* can be lower than 1 (see, for example, reference 52). This is obviously the case for self-diffusion in a molecular liquid. Theoretical studies have confirmed the validity of the Stokes-Einstein in these types of systems too^57^. The deviation of *f* from unity can also occur if the particle is non-spherical, particle aggregation occurs or there are strong intermolecular interactions, such as hydrogen-bonding^54–56^. All of these criteria are found, to some extent, in imidazolium-based ILs. Köddermann *et al*. investigated 1-ethyl-3-methylimidazolium bis(trifluoromethylsulfonyl)imide using molecular dynamics simulations^58^. They showed that this IL did not follow Stokes-Einstein behaviour when a value of *f* = 1 was used. This was attributed to regions in the molecule or aggregate where the structural relaxation time shows significant variation from the system average, known as “dynamic heterogeneities”. The values of *f* were found to be between 2/3 and 0.9. This is similar to the findings of McLaughlin, who showed that for a variety of molecular liquids, a pre-factor of 4 instead of 6 should be used in the Stokes-Einstein equation, equivalent here to setting *f* to 2/3 in Eq. 4 ^52^. For both the anion and cation in [C_2_MIM][OAc] Hall *et al*. found *f* = 0.71 and 0.58, respectively^50^. Tokuda *et al*. also investigated the value of the prefactor for a range of imidazolium-based ILs^43^. It was found that the prefactor *f* spanned from 0.55 to 0.83, with the average value of the prefactor found to be 0.65. Based on these results a value of *f* = 2/3 will be used in the following analysis, i.e. the prefactor of 6 will be replaced by 4 in the Stokes-Einstein Eq. (4).

**Figure 4:**
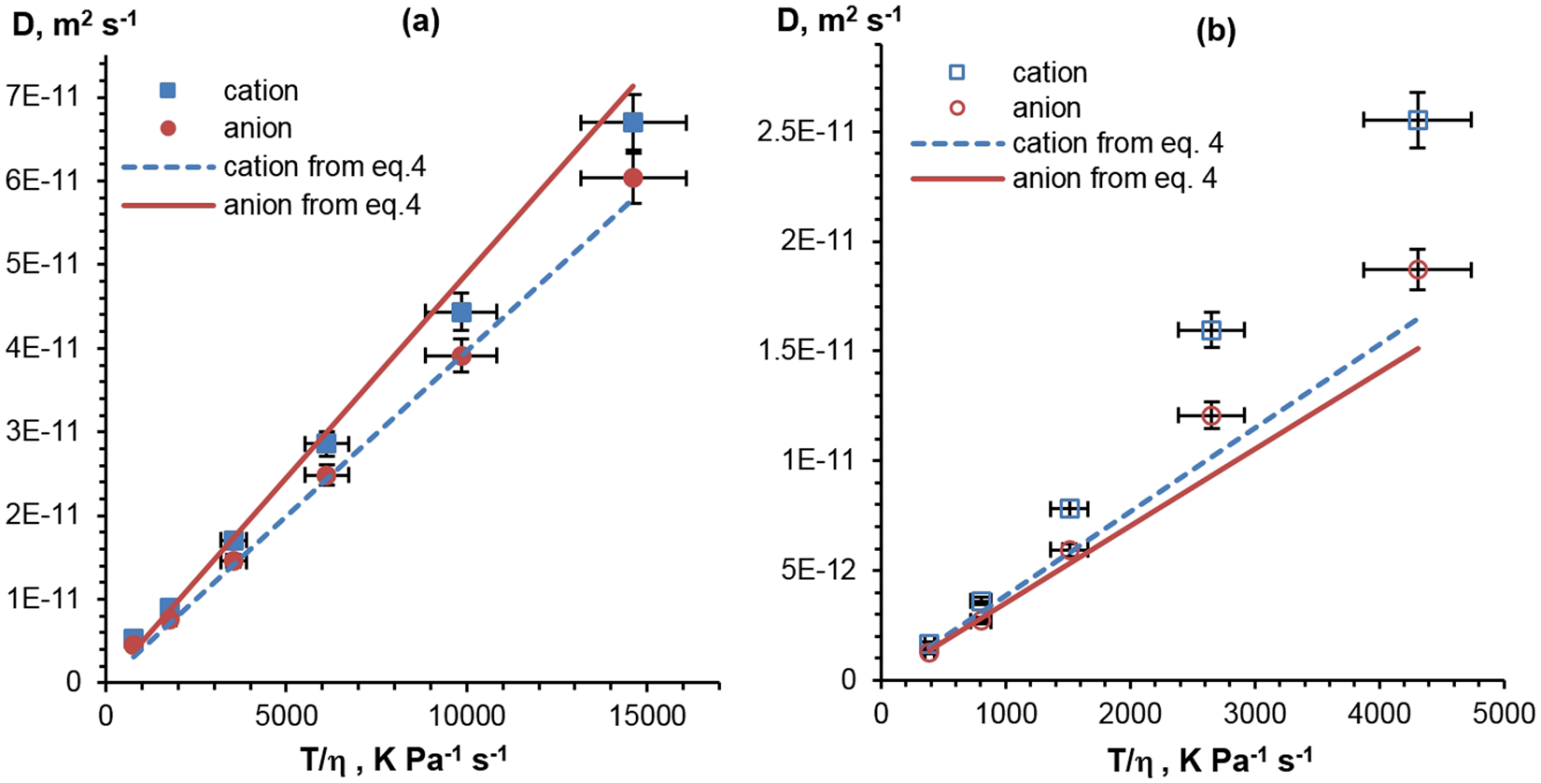
Diffusion coefficients as a function of *T/η* for [C_2_MIM][OAc] (**a**) and [C_2_MIM][OOct] (**b**). Lines show linear fits calculated with Eq. 4 and each ion’s radius from Eq. 1.

Figure 4 shows the experimental and theoretical relationships between *D* and *T/η* for both ILs. The straight lines in Fig. 4 are *no free parameter* fits, calculated directly from the Stokes-Einstein equation (Eq. 4) with *f* = 2/3 and *r*_*h,i*_ values determined from Eq. 1.

For [C_2_MIM][OAc] (Fig. 4a) the experimental data for both ions show linear dependences, suggesting that Stokes-Einstein analysis is appropriate for this system in the temperature range studied. The experimental values do not coincide with the theoretical values; the experiment shows the aforementioned anomalous diffusion, where the large cation diffuses faster than the smaller anion. From the constant gradient of experimental *D* vs *T*/*η* for both anion and cation of [C_2_MIM][OAc] (Fig. 4a) it follows that the radii of the ions have no strong temperature dependence.

For [C_2_MIM][OOct] (Fig. 4b), the data show a noticeable deviation from the lines predicted by the Stokes-Einstein equation (Eq. 4), especially at higher temperatures. This suggests a more complex situation in [C_2_MIM][OOct] than in [C_2_MIM][OAc]. The temperatures used in this study are far from the measured glass transition temperatures, *T*_*g*_, of [C_2_MIM][OAc] (*T*_*g*_ = 101 K) and [C_2_MIM][OOct] (*T*_*g*_ = 91 K), see the differential scanning calorimeter traces in the Supporting Information, Figure S7. It should be noted that the lower *T*_*g*_ of [C_2_MIM][OOct] means it is less expected to have dynamic heterogeneities across the temperature range studied here.

**Figure 5:**
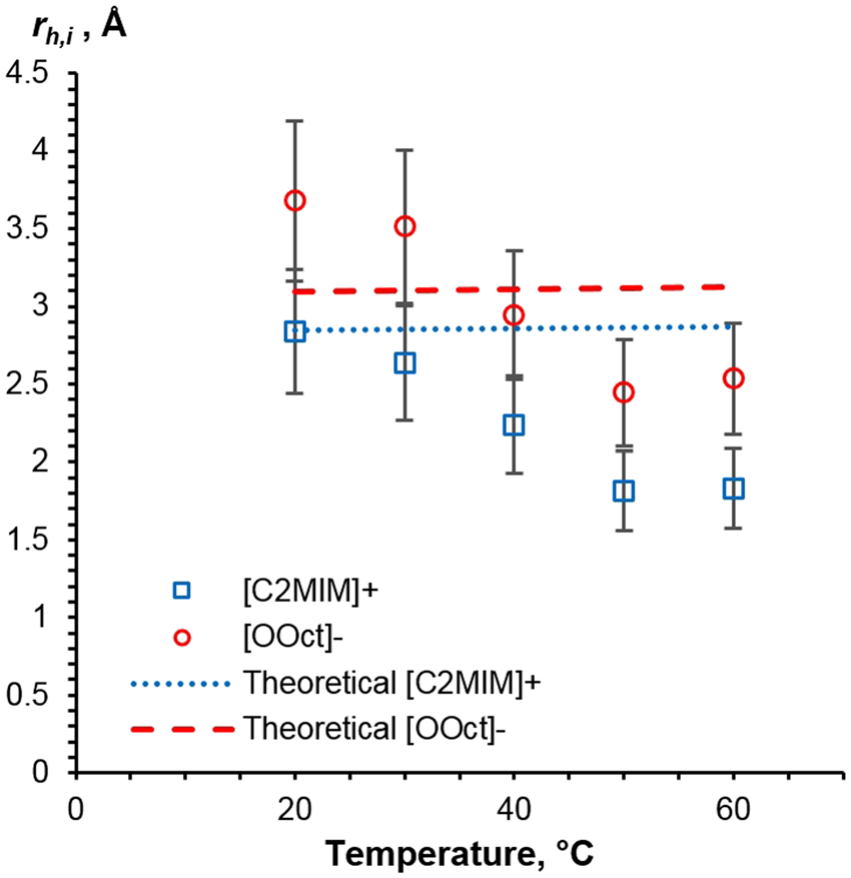
Stokes-Einstein radii of the cation and anion in [C_2_MIM][OOct] as a function of temperature (points), as follows from Fig. 4b and Eq. 4. Dashed lines correspond to cation and anion sizes calculated from Eq. 1.

Investigating this further, the influence of temperature on ionic radii for [C_2_MIM][OOct] is shown in Fig. 5. As NMR measurements can give distinct *D* values for each ion^50^, Eq. 4 was used to calculate the values for *r*_*h,i*_ from the diffusion and viscosity data at all temperatures; these will be referred to as “experimental” values. “Theoretical” *r*_*h,i*_ values were calculated directly from Eq. 1 and are shown in Fig. 5 with dashed lines. According to Eq. 1, the theoretical value of *r*_*h,i*_ is practically temperature independent as far as the dependence of density on temperature is very weak, less than 4% change across the temperature range in these ILs (see Figure S4). Both [C_2_MIM][OOct] ions’ experimental radii show a clear decrease as temperature increases. The cation experimental radii are smaller or equal to the value predicted by Eq. 1. The anion experimental radii are larger than the theoretical values at lower temperatures. This is in agreement with experimental and simulation work on other imidazolium-based ILs^50, 59^. The values given in Fig. 5 are consistent with the formation of anion-rich aggregates at lower temperatures. For example, Hou *et al*.^51^ proposed the formation of anion-cation-anion triplet in several other imidazolium-based ILs. The effective *r*_*h*_ is proportional to *M* ^*1*/*3*^ (Eq. 1), from which we can calculate the hydrodynamic radius of an anion-cation-anion triplet of 4.22 Å, with this notably being within the experimental error of the data point at 20 °C.

We suggest that a key factor that contributes to the observed decrease in anion and cation radii in [C_2_MIM][OOct] with temperature increase is the thermal instability of the ionic aggregates. A decrease in the average particle/aggregate radius at higher temperatures has already been reported for several ILs^43, 47, 51^.

### NMR Relaxometry

**Figure 6:**
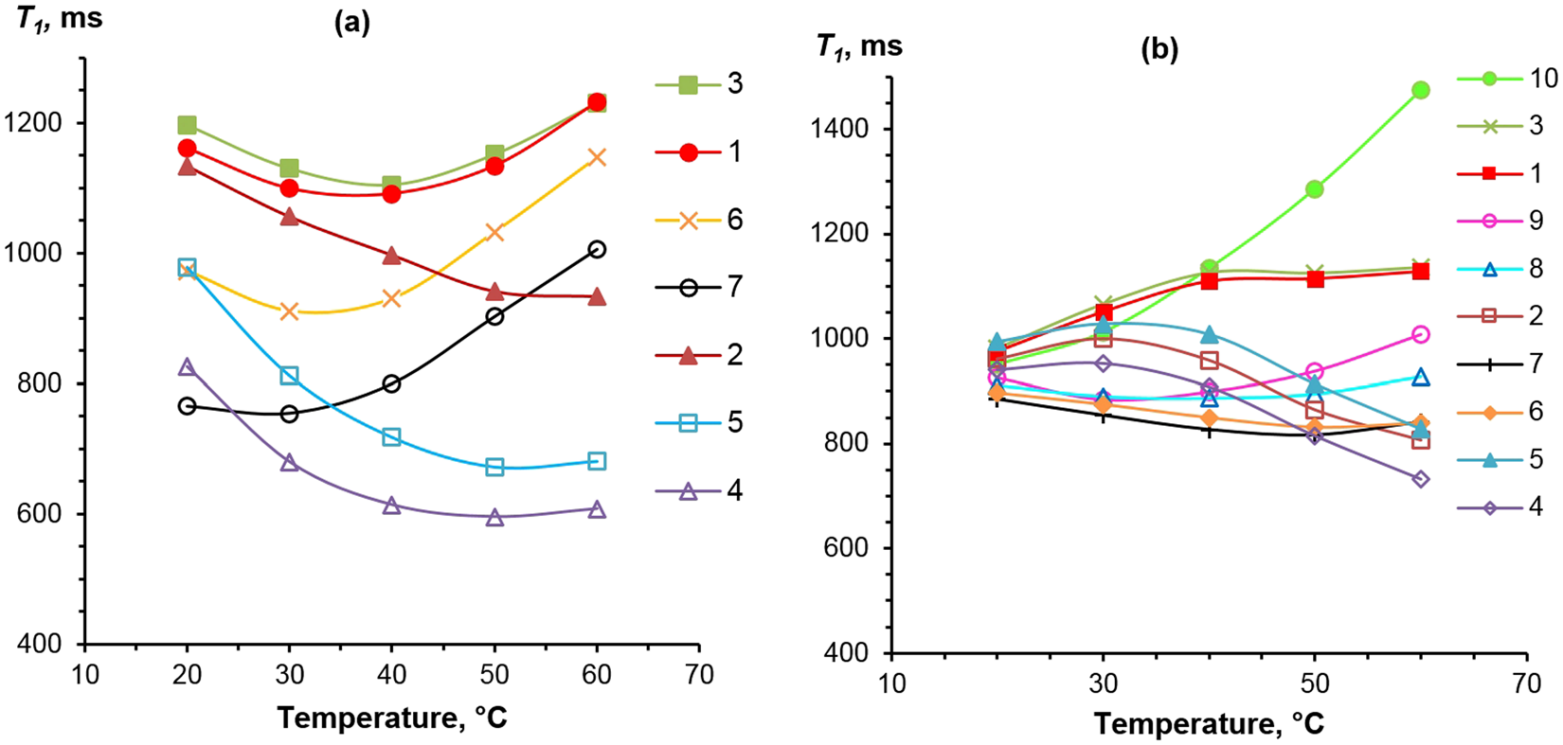
*T*_1_ relaxation times for [C_2_MIM][OAc] (**a**) and [C_2_MIM][OOct] (**b**) as a function of temperature, taken in a high-field (400 MHz) NMR spectrometer. Series numbering corresponds to the labelling shown in Fig. 1. Uncertainties of 5% are approximately the size of data points and were omitted for clarity. Lines are given to guide the eye.

Relaxation times were obtained at both high (400 MHz) and low (20 MHz) fields. In all cases the NMR relaxation signals were described with high precision fits to mono-exponential decays. Figure 6 shows the high-field NMR relaxation time *T*_1_ for [C_2_MIM][OAc] and [C_2_MIM][OOct] as a function of temperature. The *T*_1_ values for each proton resonance of [C_2_MIM][OAc] show minima between 20 °C and 60 °C. In terms of the dynamics being monitored this reveals that the magnetic fluctuation correlation times for each proton, which are responsible for the NMR relaxations, are close to that of the Larmor frequency for protons, i.e. ~1/(400 MHz). Traditionally, the terms ‘solid-like’ or ‘liquid-like’ have been used to describe NMR relaxation regimes where the system correlation times are longer or shorter than this timescale, respectively. Temperature increase leads to a *T*_1_ decrease in the ‘solid-like’ regime and an increase in the ‘liquid-like’ regime. It can therefore be concluded that on a timescale corresponding to 1/(400 MHz), [C_2_MIM][OAc] has a transition between ‘solid-like’ and ‘liquid-like’ response between 20 °C and 60 °C, with a minimum that corresponds to a correlation time of ~ 0.25 ns.

In contrast to [C_2_MIM][OAc], [C_2_MIM][OOct] shows a range of different *T*_1_ temperature dependences for different protons across the ions, matching different structural features of the two ions. As with [C_2_MIM][OAc], several of the [C_2_MIM][OOct] peaks are in the transitional regime, from ‘solid’ to ‘liquid-like’ behaviour, at the temperatures studied, specifically peaks 6, 7, 8 and 9 (recall Fig. 1). These protons are within the relatively mobile section of the anion carbon chain or on the more mobile side-chain on the cation. Peaks 2, 4 and 5 show a decreasing *T*_1_ with increasing temperature. The carbon atoms bonded to the protons corresponding to these three resonances therefore have restricted intra-molecular mobility. Peak 10 shows strong ‘liquid-like’ behaviour, with *T*_1_ increasing as temperature increases. This proton is at the end of a long, flexible carbon chain and therefore is relatively mobile as compared to the rest of the molecule.

The most interesting feature of the [C_2_MIM][OOct] *T*_1_ data is the convergence of *T*_1_ values for all 10 resonances towards a single value at lower temperatures. The NMR data was measured far above *T*_*g*_ for both these ILs (see previous section); this convergence of spin-lattice relaxation times in [C_2_MIM][OOct] is therefore unlikely to be the result of dynamic heterogeneities. Spin diffusion could equilibrate *T*_1_ across the protons within an ion, but the data indicates a shared *T*_1_ across both ions, suggesting a spatial closeness of cations and anions^60^. For *T*_1_, there are many possible relaxation mechanism that can be considered; these can be classified as either inter- or intra-ion interactions^61–64^. Inter-ion interactions give information on the ions’ diffusion^61, 62, 64, 65^, however, the diffusion coefficients of the cation and anion are known to be different in both of these ILs. Therefore, it is unlikely that diffusion and its effect on spin-lattice relaxation are the source of the unified *T*_1_ values. Additionally, at 400 MHz the spectral density is quite insensitive to diffusion. The contribution of diffusion in ILs as a spin-lattice relaxation mechanism is usually relatively insignificant at Larmor frequencies of 10 MHz and above^62^. Intra-ion interactions consist of internal rotations; the structural differences between the [C_2_MIM] cation and octanoate anion would make a common relaxation time unexpected. Indeed, at 400 MHz the *T*_1_ will be sensitive to the spectral density related to local-level bond reorientations. The dynamics will be different for each proton in the IL, as observed in [C_2_MIM][OAc]. Therefore, we suggest that the anion and cation are instead aggregating over a sufficiently long timescale to lead to a shared (through spin-diffusion/common relaxation mechanism) single *T*_*1*_ across both cation and anion. This is consistent with the presence of persistent ion-pairing at lower temperatures and, perhaps, even more complex structuring in [C_2_MIM][OOct] which was not found in [C_2_MIM][OAc]. We suggest that the breakup of this structuring is the cause for the apparent decrease in the hydrodynamic radius with temperature increase in the [C_2_MIM][OOct] system, as shown in Fig. 5.

### Low Field Relaxometry

At Larmor frequencies of 400 MHz and above, NMR relaxometry is primarily sensitive to relatively rapid motions, such as internal rotation within molecules/ions. By decreasing the field strength, slower and larger molecular rotations and molecular translations can be investigated.

The Stokes-Einstein equation can be extended to consider the rotational correlation time of a diffusing particle in a viscous medium. This relationship is described by the Stokes-Einstein-Debye equation^50, 66^,

5 $${\tau }_{rot}=\frac{4}{3}\pi {r}_{h}^{3}\frac{\eta }{{k}_{B}T}$$

where *τ*_*rot*_ is the molecular rotational correlation time and *r*_*h*_ is the radius of the particle. Classical NMR relaxation theory can be used to relate the NMR relaxation times to the molecular rotational correlation times in the simplified case of two protons at a fixed distance *r*_*H−H*_, interacting with each other via their magnetic dipolar fields. In the high-temperature regime for this simplified case, according to the work of Bloembergen, Purcell and Pound (BPP)^67^ we have,

6 $$\frac{1}{{T}_{1}}=\frac{1}{{T}_{2}}=10A{\tau }_{rot}$$

where *A* is given by the following equation,

7 $$A=\frac{3}{20}{\gamma }^{4}{\hslash }^{2}{(\frac{{\mu }_{0}}{4\pi })}^{2}{{r}_{H-H}}^{-6}$$

and *γ* is the gyromagnetic ratio for protons, *ħ* is the reduced Planck constant, *µ*_0_ is the permeability of free space and *r*_*H−H*_ is the inter-proton distance, and in our analysis this will be an effective inter-proton distance due to our system being more complicated than the model’s simple isolated spin pair approach. Therefore, combining Eqs 5 and 6 will provide an equation relating the NMR relaxation times to the macroscopic viscosity as follows,

8 $${T}_{1}={T}_{2}=\frac{3{k}_{B}}{(40\pi A{r}_{h}^{3})}\frac{T}{\eta }$$

**Figure 7:**
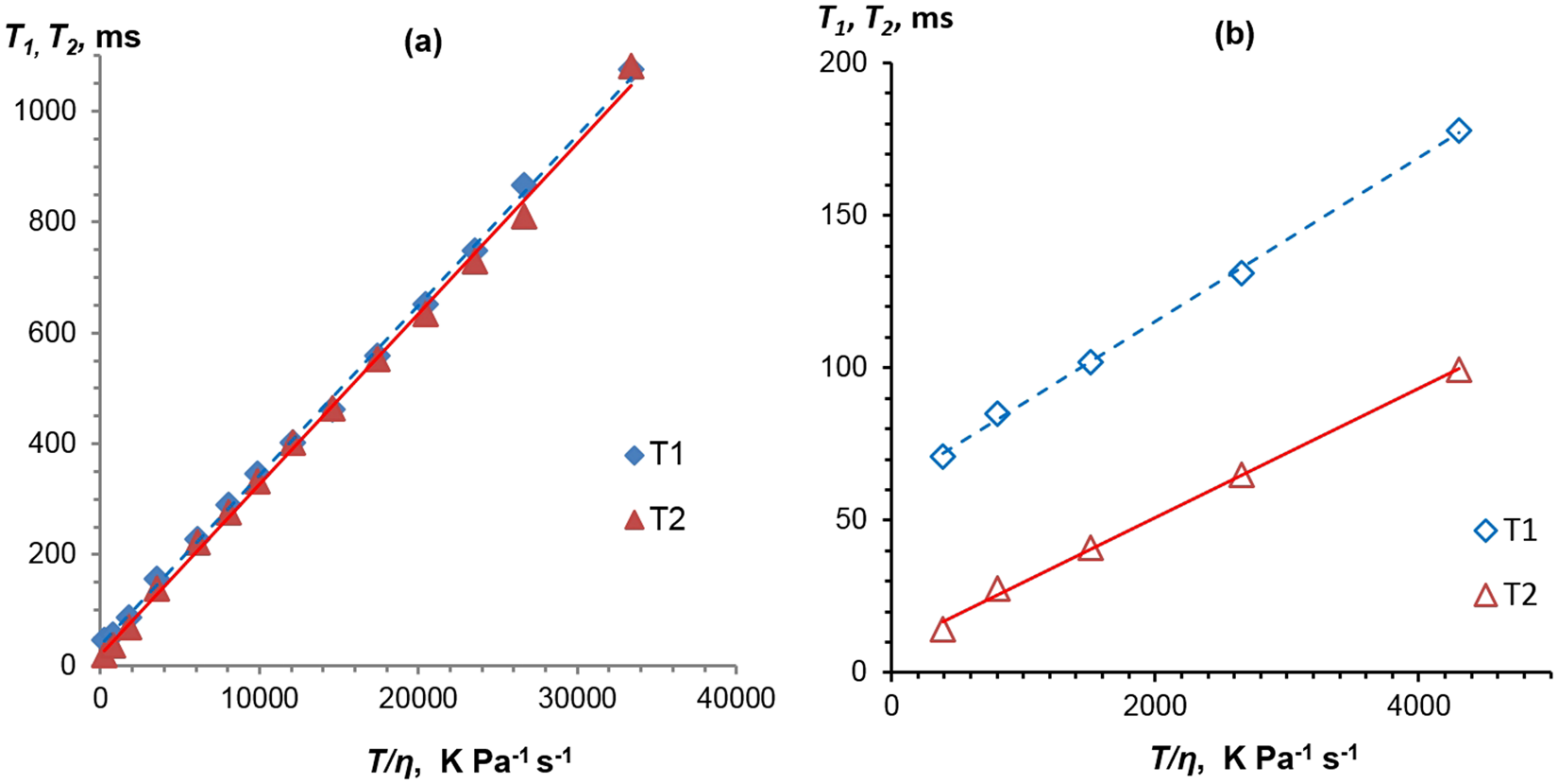
*T*_1_ and *T*_2_ dependences on *T/η* for [C_2_MIM][OAc] (**a**) and [C_2_MIM][OOct] (**b**). Solid lines are linear fits to the data.

Figure 7 shows the relationship between the low field NMR relaxation times and *T/η*. More details on BPP analysis can be found in the Supplementary Information, with fitting parameters in Table S1 and fits in Figure S5. All the data show a linear trend, as expected from Eq. 8. However, for [C_2_MIM][OOct] (Fig. 7b), the data deviates from Eq. 8, in that *T*_1_ ≠ *T*_2_ and there is a non-zero intercept for both relaxation times. The slopes were used to calculate values of *r*_*h*_. It should be noted, that the low-field experiments used here have insufficient chemical resolution to distinguish between the cation and the anion, therefore the *r*_*h*_ values in this section represent an averaged value over the two ions. The same value of *A* was used for both ILs, calculated using an estimate of *r*_*H-H*_ = 2.40 Å based on the molar ^1^H density of the ILs. This gave ion-averaged values of *r*_*h*_ = 2.88 Å for [C_2_MIM][OAc] for both *T*_1_ and *T*_2_ (Fig. 7a), and *r*_*h*_ = 3.01 Å and 3.26 Å for [C_2_MIM][OOct], from *T*_1_ and *T*_2_ data, respectively (Fig. 7b). All the *r*_*h*_ values calculated from the relaxometry are similar, though slightly higher, than those obtained from diffusion/viscosity data and the sizes obtained from Eq. 1. It is remarkable that such a simple approach gives such reasonable values to the size of the ions determined from the NMR relaxation measurements.

The two [C_2_MIM][OAc] datasets give practically identical values for *r*_*h*_, as expected from Eq. 8 (Fig. 7a). This means that [C_2_MIM][OAc] has a ‘liquid-like’ response and can be adequately described by the Stokes-Einstein-Debye equation. However, the *T*_1_ and *T*_2_ gradients for [C_2_MIM][OOct] are different to each other, leading to slightly different *r*_*h*_ values (Fig. 7b). This means that [C_2_MIM][OOct] cannot be considered to be in the high-temperature regime at the temperature range studied here (20–60 °C). This suggests that [C_2_MIM][OOct] is near to the ‘solid-like’ regime at ambient temperatures on a timescale set by 1/(20 MHz) Larmor frequency. Furthermore, in [C_2_MIM][OOct] there is an expected spectrum of relaxation times, due to *i)* the presence of the long ‘tail’ on the anion and *ii)* the higher degree of structuring we are proposing in [C_2_MIM][OOct]. Both of these will introduce a significant degree of slower relaxation modes; unlike *T*_1_, *T*_2_ is sensitive to the ‘zero frequency’ dynamics, or spectral density *J(0)*, of a system^67^. Therefore these slower modes of relaxation will cause *T*_2_ to have a different value to that of *T*_1_, as observed in Fig. 7b.

## Discussion

From the data presented above it is clear that [C_2_MIM][OOct] behaves differently from [C_2_MIM][OAc] at a molecular level. While this is not surprising in itself (long octanoate tail vs. small acetate ion), the cause and effects of these differences may shed some light on the properties of these systems. It is well known that pure ILs tend to associate and form ion pairs or even larger aggregate structures^43, 48, 51, 68, 69^. In the section concerning self-diffusion, two types of “anomalies” were discussed: *i)* slower diffusion of small anions compared to the larger cations in [C_2_MIM][OAc] and *ii)* slower than predicted diffusion of the anion in [C_2_MIM][OOct]. In both cases, we suggest that the main reason is the formation of multi-ion aggregates in agreement with Hou *et al*.^51^ However, the aggregates in [C_2_MIM][OAc] and [C_2_MIM][OOct] are different. We hypothesise that in [C_2_MIM][OAc] the ions form small, stable anion-rich aggregates, while in [C_2_MIM][OOct] the aggregates are larger but can break up, at least partially, at increased temperatures and under flow. The evidence supporting this hypothesis is given below.

Figure 3 shows that the viscosity of [C_2_MIM][OOct] is significantly larger than that of [C_2_MIM][OAc]. Lower diffusivity and higher viscosity of [C_2_MIM][OOct] is expected purely from a molecular weight or size argument. However, the molecular weight of [C_2_MIM][OOct] is only 1.5 times that of [C_2_MIM][OAc], whereas the viscosity is between 4.6 (20 °C) and 3.4 (60 °C) times that for [C_2_MIM][OOct] at the corresponding temperatures. This suggests the [C_2_MIM][OOct] forms aggregates at lower temperatures and that these break up as temperature increases. This is demonstrated in Fig. 5 which shows the decrease of effective anion and cation size with increasing temperature.

The activation energies shown in Table 1 also support this picture. The closeness of *E*_*a,D*_ and *E*_*a,η*_ for [C_2_MIM][OAc] means that the energy barrier to diffusion is the same as that to flow, suggesting that the effective size of the ions is the same in diffusion and flow. In contrast, in [C_2_MIM][OOct] *E*_*a,η*_ is substantially lower than *E*_*a,D*_, suggesting that different sized entities are responsible for diffusion and flow.

As well as direct comparison of viscosity and diffusivity, these two different datasets can be combined, revealing more information. The decrease of effective cation and anion radii with temperature for [C_2_MIM][OOct], as shown in Fig. 5, suggests entropic effects. As stated previously, this is consistent with the breakup of large aggregates into smaller constituents at higher temperatures and matches the observed pattern in the viscosity behaviour. The absence of this decrease of *r*_*h,i*_ in [C_2_MIM][OAc] fits with the picture of smaller and shorter-lived aggregates. This is supported by [C_2_MIM][OOct] high-field *T*_1_ data, where the convergence of *T*_1_ values towards a single value at low temperatures suggests the formation of aggregates^47^. The aggregates in terms of their dynamics will have a cooperative global correlation time giving rise to an effective combined single NMR relaxation time. As the temperature increases, the aggregates will diffuse faster and entropic forces will cause them to break up, which in turn will enable each ion to move independently, giving rise to different measured NMR relaxation times for each ion / resonance. It is interesting to note that this convergence is strongest at 20 °C to 30 °C, correlating well with the temperatures where the [OOct]^−^ ion has a larger effective hydrodynamic radius than Eq. 1 predicts (Fig. 5). Additionally, unlike [C_2_MIM][OAc], [C_2_MIM][OOct] does not follow the Stokes-Einstein-Debye equation, as shown in Fig. 7. This suggests a large correlated system with slow dynamical motion and is consistent with the aggregation in [C_2_MIM][OOct] that we propose here.

## Conclusions

The physical properties of two imidazolium-based ionic liquids, [C_2_MIM][OAc] and [C_2_MIM][OOct], were investigated using NMR and rheological measurements. NMR diffusion coefficients and relaxation times at two field strengths are accompanied by viscosities for both ILs at temperatures from 20 to 60 °C.

The diffusion data confirmed the presence of ‘anomalous’ diffusion in [C_2_MIM][OAc] and likewise showed that in [C_2_MIM][OOct] the anion diffuses slower than expected. In both cases, aggregation of anions was hypothesised. The activation energy deduced from viscosity-temperature and diffusion-temperature data suggested ionic aggregation in [C_2_MIM][OOct] with aggregates breaking under flow and with increasing temperature, contrary to more stable anionic aggregates in [C_2_MIM][OAc]. NMR relaxometry complemented the diffusion and viscosity data, supporting the different types of aggregation in each IL. The results obtained provide some groundwork for an understanding of the influence of anion chain length on the dissolution of cellulose in these ILs.

## Materials and Methods

### Materials

[C_2_MIM][OAc] was obtained from Sigma Aldrich with a purity of >97% for NMR measurements. [C_2_MIM][OOct] was purchased from IoLiTec GmbH, Germany, with a purity of >95%. The high-purity fluids used for NMR measurements were both used as received and stored under a dry nitrogen atmosphere with no exposure to atmospheric humidity. Water content in [C_2_MIM][OAc] was measured by Karl-Fischer titration and monitored *in situ* by NMR. It was found to conform to the specification of <0.5% water by weight in all experiments^13^. The [C_2_MIM][OOct] was found to have water content of <0.2 wt% by Karl-Fischer and remained below 0.5 wt% during NMR experiments.

### NMR Sample Preparation

All NMR samples were prepared in a glove box, under a nitrogen atmosphere. The dew point level of the glove box was maintained between −40 and −70 °C. The samples were prepared according to the recommendations of Annat *et al*.^70^, such as keeping the sample height short (below 1 cm) to minimise convection currents. The sample tubes were permanently sealed to minimise water contamination from atmospheric humidity.

### NMR Diffusion

Self-diffusion coefficients, *D*, were measured on a Bruker Avance II 400 MHz spectrometer with a diffusion probe (Diff50). Gradient strengths of up to 20 T m^−1^ were obtainable and the gradient field strength was calibrated by measuring the self-diffusion coefficient of water at 20 °C. A pulsed-field gradient (PFG) technique was used, with a stimulated echo pulse sequence and bipolar gradients^70^. The diffusion time, *Δ*, was 100 ms for all measurements. Additionally in a separate set of experiments this parameter was varied from 50 ms to 200 ms to confirm the stability of the determined self-diffusion coefficients. Temperature was controlled with the accuracy of ±0.1 °C. The signal attenuation as a function of gradient strength was fitted using the Stejskal-Tanner equation to find *D* ^71^, an example fit is shown in Figure S6 in the Supplementary Information. Diffusion coefficients for each ion were averaged over the relevant peaks for the cation (peaks 1–5, 7) and the anion (6, 8-10), based on the spectral assignment of each peak, see Fig. 1. In order to minimise convection currents, the water-cooling system was maintained at 15 °C below the desired sample temperature. The uncertainty in the averaged diffusion coefficient values is approximately 3%, based on the standard deviation of the diffusion coefficient values for each of the imidazolium cation resonances.

### NMR Relaxometry

Low-field relaxometry measurements were carried out on a 20 MHz Maran Benchtop NMR spectrometer. The spin-lattice relaxation time, *T*_1_, was measured using an inversion-recovery pulse sequence and the spin-spin relaxation time, *T*_2_, was measured using the Carr-Purcell-Meiboom-Gill (CPMG) pulse sequence. Temperatures were maintained with a heating element and a dry airflow at 30 °C and above. Temperatures at 20 °C and below were reached using a cryogenic nitrogen flow. In both cases the error was ±0.2 °C.

High-field relaxometry measurements were carried out on a Bruker Avance II 400 MHz spectrometer with a diffusion probe (Diff50). The pulse sequences used at 400 MHz were the same as those used at 20 MHz. Temperatures were maintained using a heating element and a dry airflow. In all cases, samples were left for 10 mins to equilibrate before each experiment.

### Viscosity

Steady state viscosity of the [C_2_MIM][OOct] was measured using a stress-controlled Bohlin Gemini rheometer, equipped with a cone-plate geometry (4°–40 mm) and a Peltier temperature control system. To prevent water uptake from the air, a thin film of low-viscosity silicon oil was used to cover the edges of the measuring cell^14^. Viscosity-shear rate dependence of each IL was measured at the same range of temperatures used in the NMR study. Viscosity data for [C_2_MIM][OAc] are taken from reference 14, using identical equipment and methods.

### Density

Density was measured for both ILs over a temperature range of 20–80 °C using an Anton Paar DMA 4100 M density meter. The values varied between 1.10–1.07 g cm^−3^ for [C_2_MIM][OAc] and 1.00–0.96 g cm^−3^ for [C_2_MIM][OOct]. These agree with literature values for [C_2_MIM][OAc]^14, 49^. When relevant, exact density values are used at the corresponding temperatures. Full density data are given in Figure S2, in the Supplementary Information.

### Glass Transition Temperatures

The glass transition temperature was measured for both ILs on a TA Q2000 Differential Scanning Calorimeter (DSC), with a liquid nitrogen cooling system for temperature control. The temperature and heat flow of the system were calibrated using standard samples of indium and adamantane. Samples were placed in aluminium hermetically sealed pans and measured at a rate of 10 K min^−1^ from −130 °C to 30 °C.

## Electronic supplementary material

Supplementary Information
