# Supplementary material for: NMR and Rheological Study of Anion Size Influence on the Properties of Two Imidazolium-based Ionic Liquids
Source: Sci Rep. 2017 Aug 21;7:8968. doi: 10.1038/s41598-017-09509-2 (PMC5566215; doi:10.1038/s41598-017-09509-2)
Supplement: Supplementary file 1 — Supplementary Information [file 41598_2017_9509_MOESM1_ESM.pdf]

SUPPLEMENTARY INFORMATION

NMR and Rheological Study of Anion Size Influence on the Properties of Two Imidazolium-based Ionic Liquids

Stephen GREEN[1], Michael E. RIES[1]*, Jamie MOFFAT[2], Tatiana BUDTOVA[3]*

1 - Soft Matter Physics Research Group, School of Physics and Astronomy, University of Leeds, Leeds, LS2 9JT, United Kingdom

2 - Innovia Films R&D Centre, West Road, Wigton, Cumbria CA7 9XX, United Kingdom

3 - MINES ParisTech, PSL Research University, Centre for Material Forming (CEMEF), UMR CNRS 7635, CS 10207, 06904 Sophia Antipolis Cedex, France

The data for this article can be found at http://doi.org/10.5518/122.

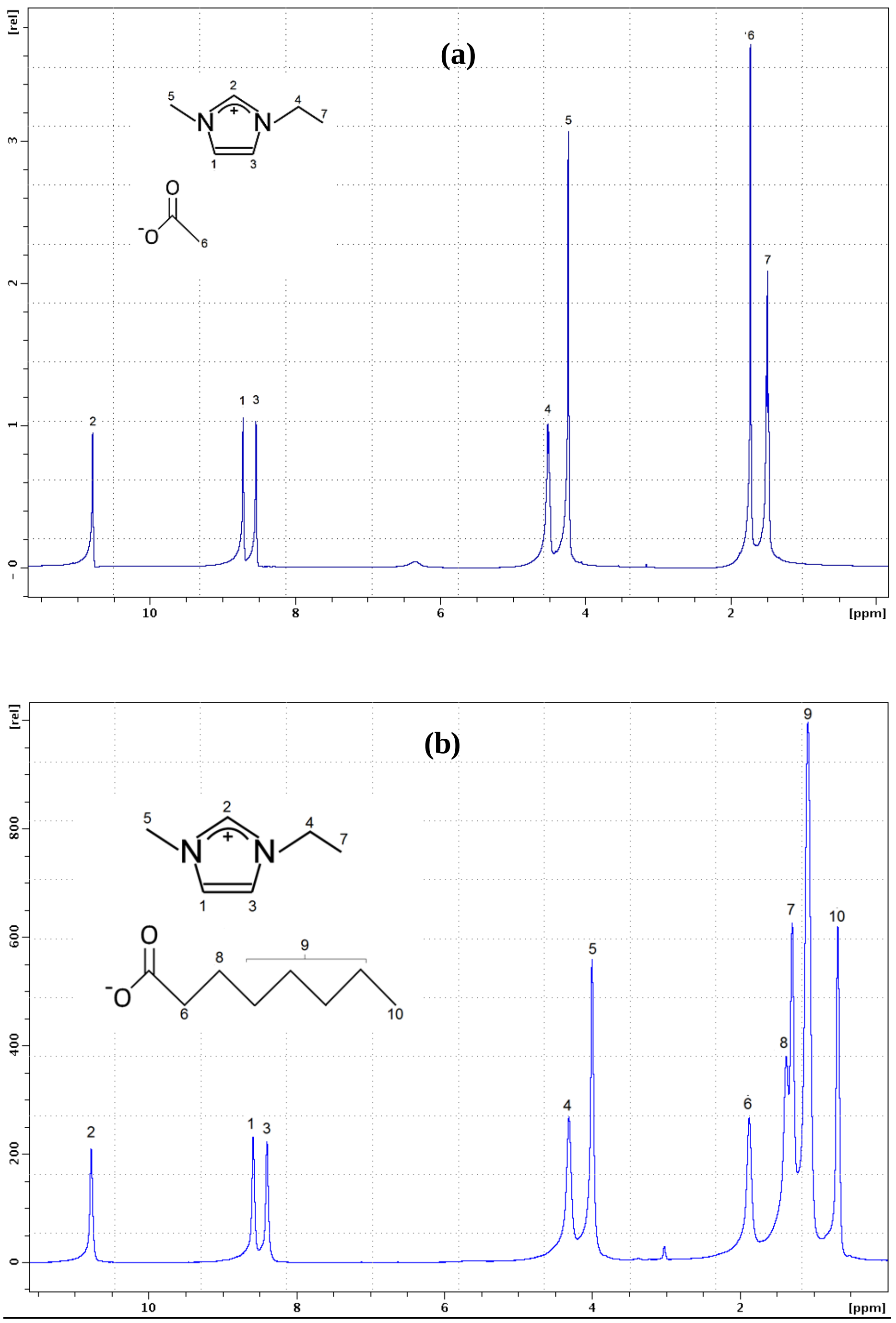

Figure S1.

^{1}H NMR spectra of [C_2MIM][OAc] (a) and [C_2MIM][OOct] (b) at 20 ºC. The chemical structure is shown with the inset and peaks are labelled accordingly.

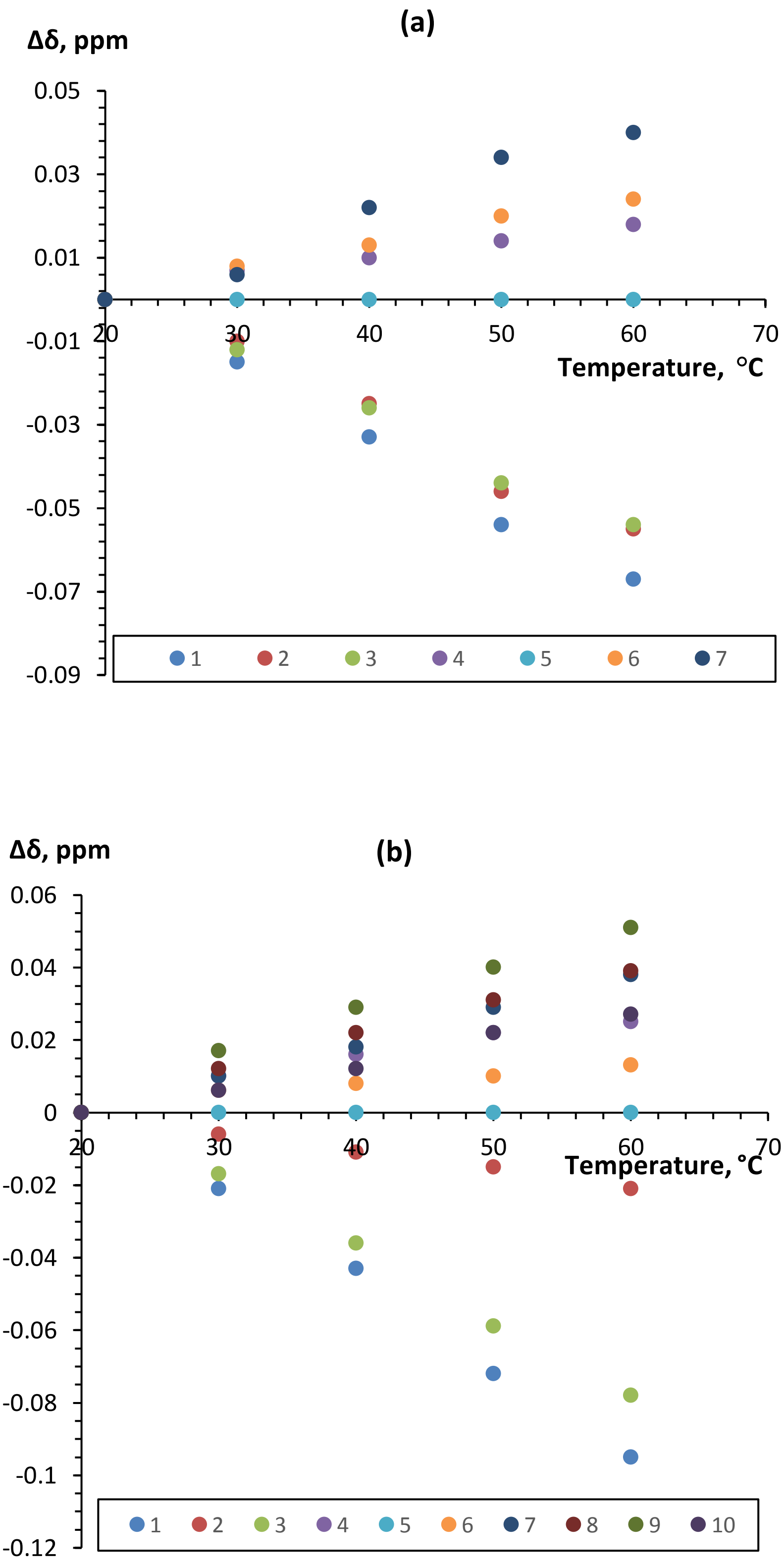

Figure S2

Chemical shift as a function of temperature for [C_2MIM][OAc] (a) and [C_2MIM][OOct] (b). Resonances are numbered according to Figure 1, see also Figures S1a and S1b, respectively.

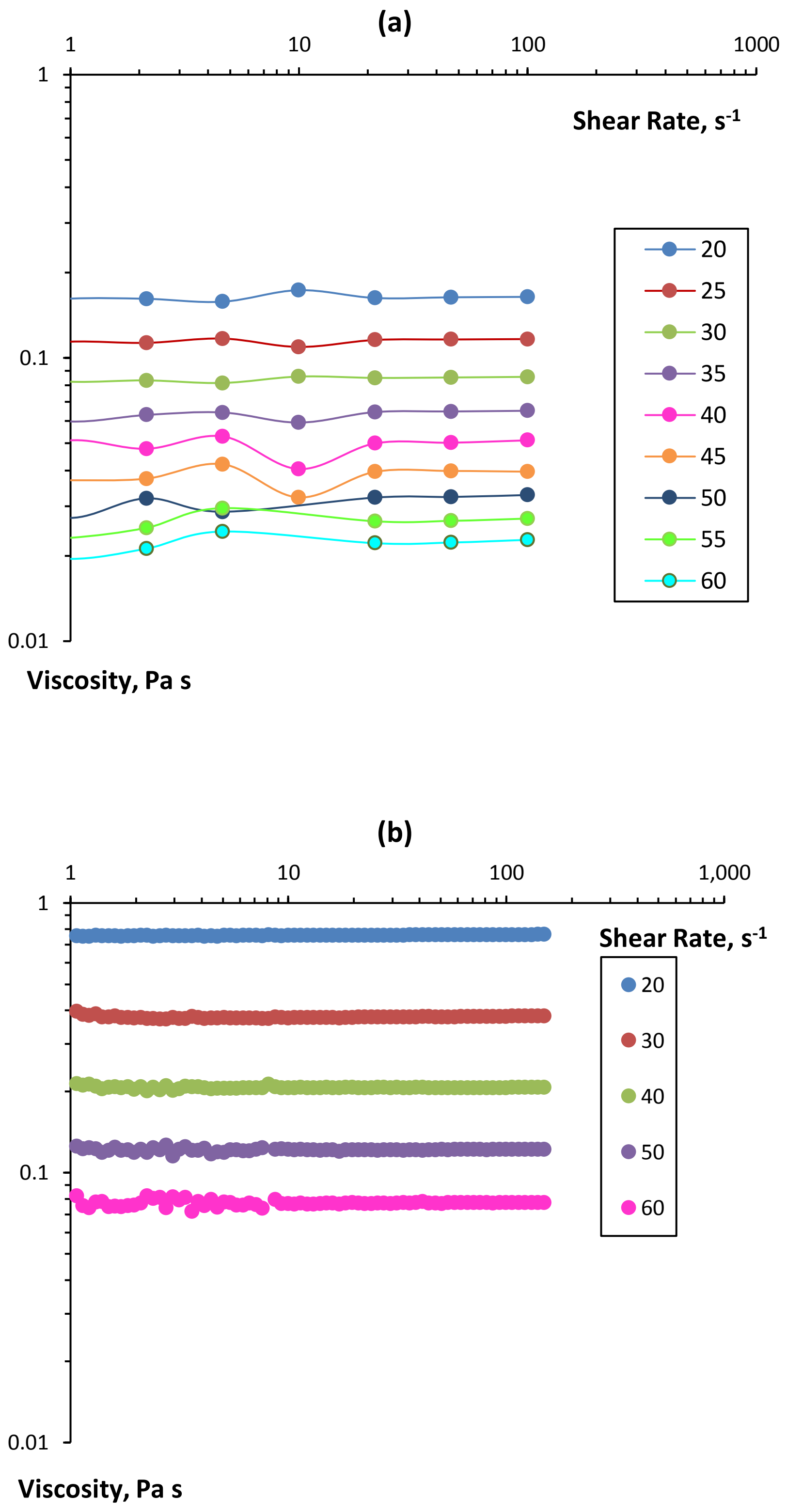

Figure S3

Steady-state viscosity as a function of shear rate for [C_2MIM][OAc] (a) and [C_2MIM][OOct] (b).

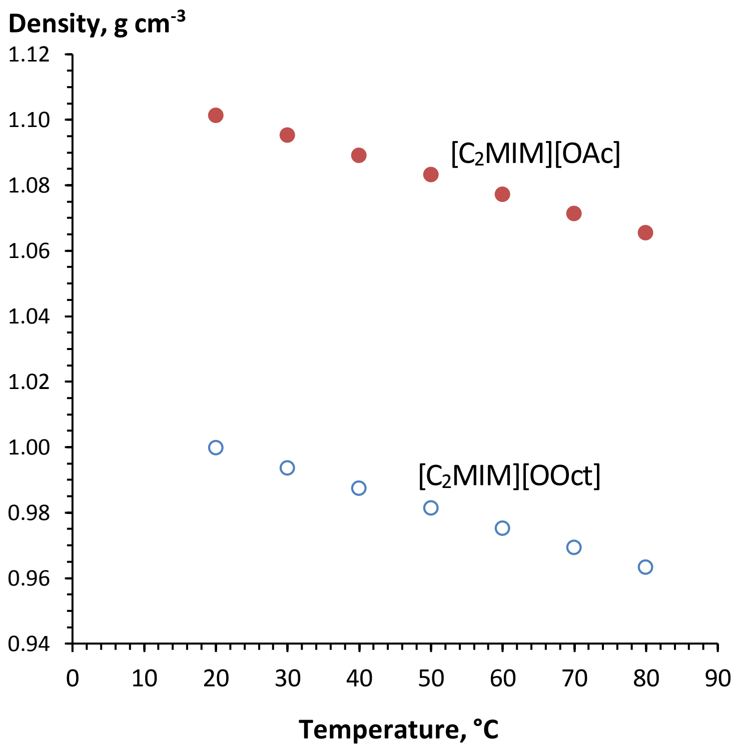

Figure S4

Density as a function of temperature for [C_2MIM][OAc] and [C_2MIM][OOct].

Table S1. Fitting parameters for relaxation data, based on BPP theory [1]

	$[C_2MIM][OAc]$	$[C_2MIM][OOct]$: T_1	$[C_2MIM][OOct]$: T_2
A / s^{-2}	9.50×10^{8}	6.69×10^{8}	6.69×10^{8}
$r_{H\text{-}H}$ / Å	2.12	2.25	2.25
E_a / kJ mol^{-1}	38.4	21.6	43.5
τ_0 / s	2.37×10^{-16}	3.94×10^{-13}	1.56×10^{-16}

Low-field relaxometry measurements were carried out on a 20 MHz Maran Benchtop NMR spectrometer. Figure S5 shows the low-field relaxation times for $[C_2MIM][OAc]$ and $[C_2MIM][OOct]$ as a function of temperature. T_1 and T_2 increase as temperature increases for both ILs. This type of behaviour is predicted by Bloembergen, Purcell, and Pound (BPP) theory [1]. BPP theory can be used to relate the NMR relaxation times to the molecular rotational correlation times in the simplified case of two protons at a fixed distance $r_{H\text{-}H}$ apart, interacting with each other via their dipolar fields as follows,

$$R_1 = \frac{1}{T_1} = 2A\left(\frac{\tau}{1+\omega^2\tau^2} + \frac{4\tau}{1+4\omega^2\tau^2}\right)$$

$$R_2 = \frac{1}{T_2} = A\left(3\tau + \frac{5\tau}{1+\omega^2\tau^2} + \frac{2\tau}{1+4\omega^2\tau^2}\right)$$

$$A = \frac{3}{20}\gamma^4\hbar^2\left(\frac{\mu_0}{4\pi}\right)^2 {r_{H-H}}^{-6}$$

where τ is the correlation time of the molecular rotation, ω is the Larmor frequency, γ is the gyromagnetic ratio for protons, $\hbar$ is the reduced Planck constant, μ_0 is the permeability of free space and $r_{H\text{-}H}$ is the inter-proton distance.

The T_1 behaviour indicates that at the 1/(20 MHz) timescale, both ILs are in a 'liquid-like' state at the upper end of the temperature range studied. However, the T_1 values for $[C_2MIM][OOct]$ show a turning point at around 0 °C. This turning point indicates the transition from the 'liquid-like' regime to the 'solid-like' regime, on the 20 MHz timescale. The dashed lines in Figure S5 show theoretical fits, obtained from BPP theory [1]. The correlation time was modelled using a simple Arrhenius-type equation, as shown below,

$$ln\tau = ln\tau_0 + \frac{E_a}{RT}$$

where τ_0 is the correlation time at "infinite temperature", E_a is activation energy for molecular rotation, R is the molar gas constant and T is the temperature. Typical values for the correlation times are ~10^{-9} s.

The data for [C_2MIM][Ac] are modelled reasonably well by classical BPP theory at most temperatures studied and the fitting parameters are given in Table S1. It is noteworthy that the distance between protons, $r_{H\text{-}H}$, is close to the expected value based on the density and molar volume of the ILs.

The fits for [C_2MIM][Ac], shown in Figure S5a, indicate that the dynamics of [C_2MIM][Ac] are relatively simple at these timescales, with a single correlation time across the system. Conversely, the [C_2MIM][OOct] data is not described well by classical BPP theory. It was not possible to simultaneously fit both T_1 and T_2 with a single correlation time.

The fits shown in Figure S5b were obtained by separately fitting the T_1 and T_2 datasets, indicating that the two NMR relaxation processes were described by two different correlation times. T_2 is more sensitive than T_1 to slower dynamics. This suggests there is a significant presence of these slower processes in [C_2MIM][OOct] than in [C_2MIM][Ac], which could be explained by the more complex dynamics stemming from the formation of larger aggregates, as suggested in the main article.

Relaxation Data Fitting

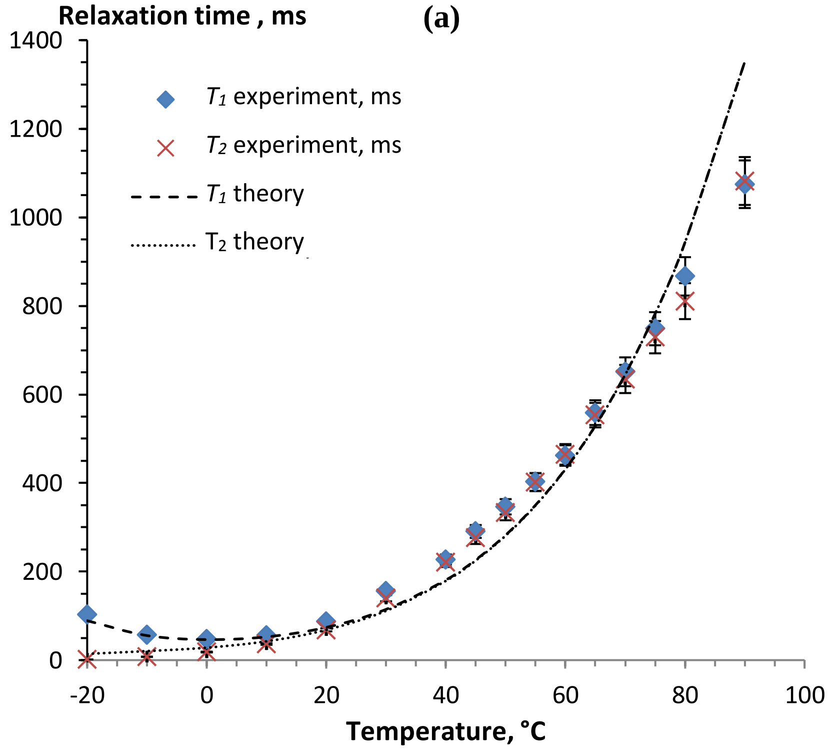

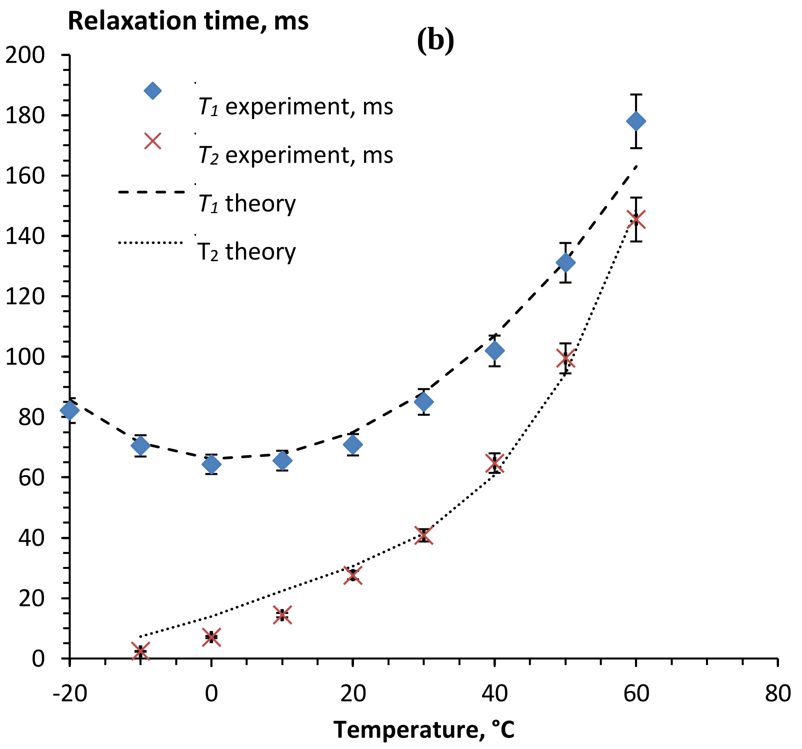

Figure S5

T_1 and T_2 relaxation times for [C_2MIM][Ac] (a) and [C_2MIM][OOct] (b) as a function of temperature, taken on a low-field (20 MHz) NMR spectrometer.

Diffusion coefficients for the ILs were measured using pulsed field gradient (PFG) NMR, as detailed in the main article. Figure S6 shows a plot of signal intensity as a function of gradient field strength for a single resonance in [C_2MIM][OOct] at 30 °C. Each resonance was fitted in the same way for both ILs and all temperatures. Figure S6 exhibits a very high quality of fit, typical of all the experiments, thus there are no indications of restricted diffusion or the need for more than one self-diffusion coefficient per resonance.

All PFG NMR experiments presented in the main article were undertaken with a diffusion time, Δ¸ of 100 ms. Additional experiments were undertaken, varying Δ between 50 ms and 200 ms. The diffusion coefficients found were the same for all Δ values used, within experimental error of <5%.

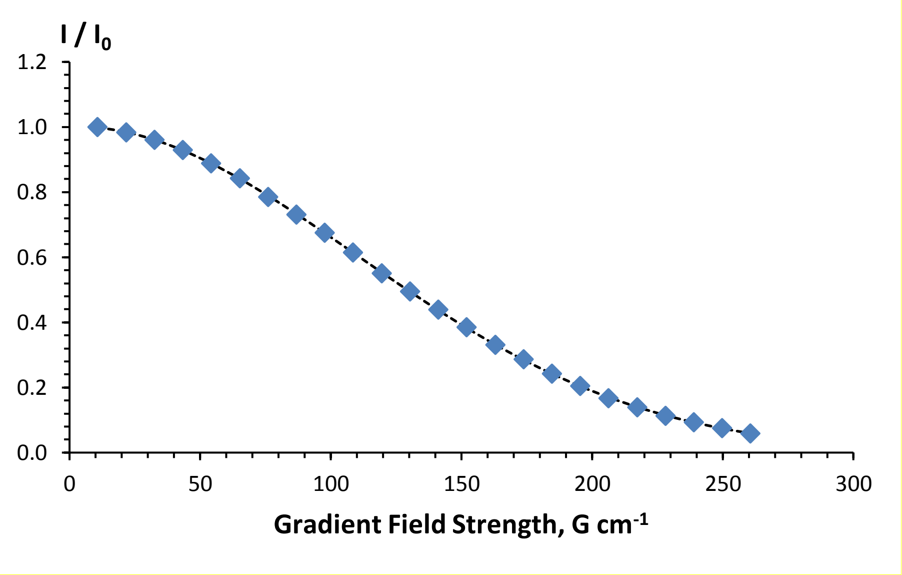

Figure S6

Example fit of signal intensity and gradient field strength in a PFG NMR experiment on [C_2MIM][OOct] at 30 °C. Points show experimental data and dashed line shows a Gaussian fit, based on the Stejskal-Tanner equation [2].

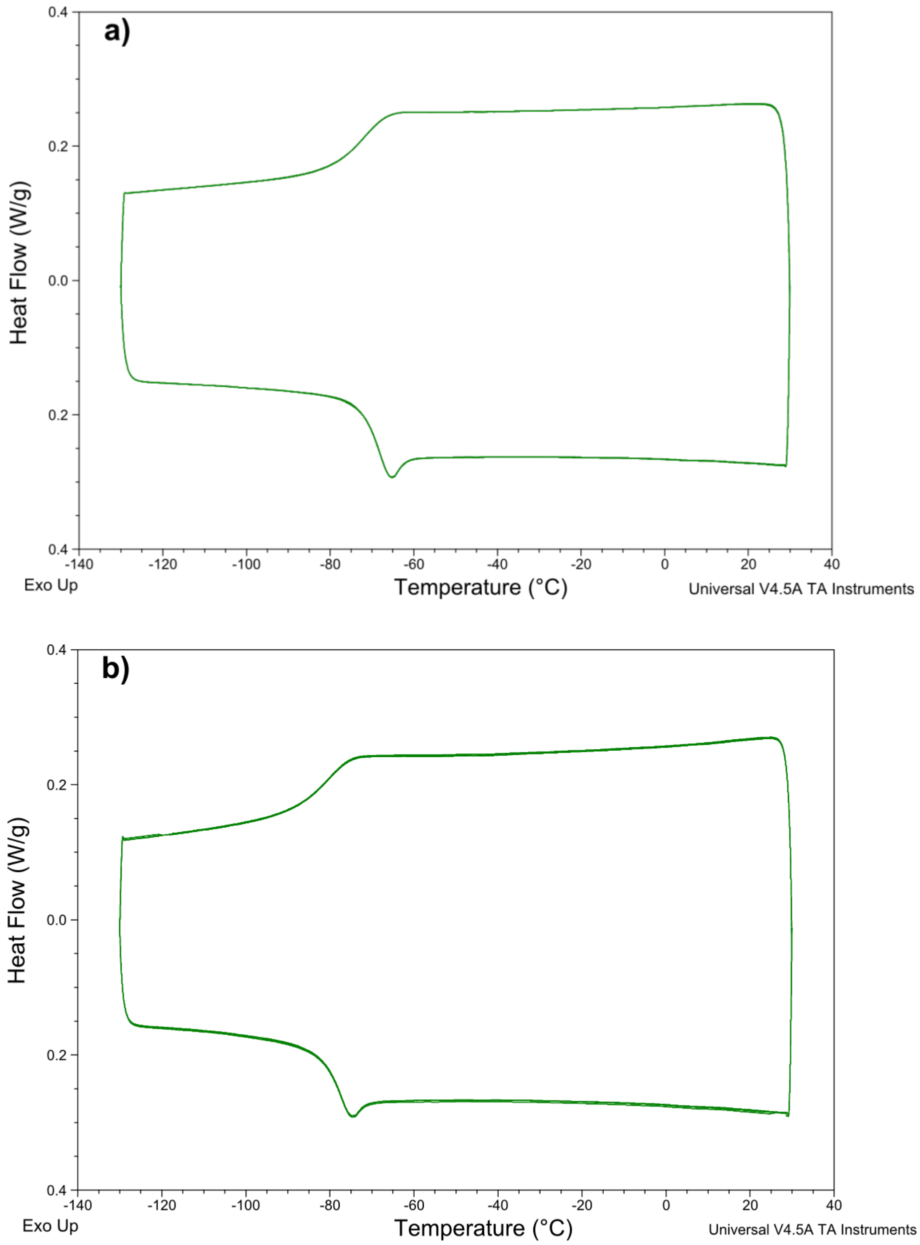

Figure S7

DSC traces of **a)** [C_2MIM][OAc] and **b)** [C_2MIM][OOct], showing the glass transitions at temperatures below – 60 °C. See main article for further details.

References

1 Bloembergen, N., Purcell, E. M. & Pound, R. V. Relaxation Effects in Nuclear Magnetic Resonance Absorption. *Physical Review* **73**, 679-712, doi:Doi 10.1103/Physrev.73.679 (1948).

2 Stejskal, E. O. & Tanner, J. E. Spin Diffusion Measurements: Spin Echoes in the Presence of a Time-Dependent Field Gradient. *The Journal of chemical physics* **42**, 288-292, doi:10.1063/1.1695690 (1965).
